# Nanomaterials targeting macrophages in sepsis: A promising approach for sepsis management

**DOI:** 10.3389/fimmu.2022.1026173

**Published:** 2022-12-09

**Authors:** Chaoying Song, Jiqian Xu, Chenggang Gao, Wanying Zhang, Xiangzhi Fang, You Shang

**Affiliations:** Department of Critical Care Medicine, Union Hospital, Tongji Medical College, Huazhong University of Science and Technology, Wuhan, China

**Keywords:** sepsis, nanomaterials, macrophages, nanotargeted therapy, nanodiagnosis

## Abstract

Sepsis is a life-threatening organ dysfunction resulting from dysregulated host responses to infection. Macrophages play significant roles in host against pathogens and the immunopathogenesis of sepsis, such as phagocytosis of pathogens, secretion of cytokines, and phenotype reprogramming. However, the rapid progression of sepsis impairs macrophage function, and conventional antimicrobial and supportive treatment are not sufficient to restore dysregulated macrophages roles. Nanoparticles own unique physicochemical properties, surface functions, localized surface plasmon resonance phenomenon, passive targeting *in vivo*, good biocompatibility and biodegradability, are accessible for biomedical applications. Once into the body, NPs are recognized by host immune system. Macrophages are phagocytes in innate immunity dedicated to the recognition of foreign substances, including nanoparticles, with which an immune response subsequently occurs. Various design strategies, such as surface functionalization, have been implemented to manipulate the recognition of nanoparticles by monocytes/macrophages, and engulfed by them to regulate their function in sepsis, compensating for the shortcomings of sepsis traditional methods. The review summarizes the mechanism of nanomaterials targeting macrophages and recent advances in nanomedicine targeting macrophages in sepsis, which provides good insight for exploring macrophage-based nano-management in sepsis.

## Introduction

Sepsis is a life-threatening organ dysfunction associated with dysregulated host response to infection (1, 2). Sepsis kills as many as one in four similar to acute myocardial infarction, stroke, or multiple injuries and ranks third in diverse disease mortality (1, 2). There are approximately 48.9 million sepsis patients worldwide, with a mortality rate of 19.7% (3). However, due to its complex pathogenesis, targeted therapies still need to be explored (2).

Monocytes/macrophages are important players in the pathogenesis of sepsis (4, 5). Induced by pathogens and cytokines in the environment, macrophages differentiate to diverse functional phenotypes and perform different functions, including killing of pathogenic microorganisms, cytokine and chemokine production (6). During sepsis, the complicated pathogenesis brings changes in macrophage function, which include phenotype reprogramming, alterations in inflammatory signaling pathways, macrophage overactivation causes inflammatory factor storm, *etc* (7, 8). These changes complicate the pathophysiological status of clinical patients, leading to the dramatically reduced effects of conventional gene therapy and drugs (9–14).

Nanomaterials are a class of materials consisting of organic or/and inorganic particles with a size of about 1 to 100 nm, and the representative classes of nanomaterials includes polymeric materials, liposomes, biomimetic materials, exosomes and metal/inorganic materials and so on (15), it has been considered a promising tool in sepsis treatment. Nanomaterials can be used as drug carriers transport inflammation-modifying drugs, because it can enhance drug targeting delivery and bioavailability, modulating pro-/anti-inflammatory roles (16). For macrophage, nanomaterials act as contrast and diagnostic devices that can detect the physicochemical properties of macrophage phagosomes and realize macrophage labelling, imaging, and long-term follow-up (17–22), the features of nanomaterials may help to improve the diagnostic and therapeutic techniques for patients with sepsis, by such as targeting macrophage activation, modulating inflammatory pathways, reprogramming macrophage polarization, *etc* (23–26). However, their characteristics and functional mechanisms targeting macrophages during sepsis have not been addressed fully in sepsis (16, 27). Herein, we review the following: i) the rationality of nanomaterials targeting macrophages; ii) the mechanisms for nanomaterials or technologies targeting sepsis-associated macrophages; and iii) the prospects of nanomaterials for the diagnosis and management of sepsis.

## The dysregulated function of macrophages in sepsis

During early sepsis, lipopolysaccharides (LPS) is recognized by toll-like receptor 4 (TLR4) of macrophages, which activate the nuclear factor-κB (NF-κB) pathway and mitogen-activated protein kinase (MAPK) pathway, causing inflammation-active mediators (such as IL-1, IL-6, IL-18, TNF-α) releasing and facilitating the clearance of pathogenic microorganisms (28). However, excessive activated macrophages cause a cascade of amplified inflammatory responses, such as “cytokine storm”, impairing host immune function and mediating organ dysfunction (29). Meanwhile, macrophage reprogramming, also known as LPS tolerance, causes a reduction in the ability of macrophage to release pro-inflammatory cytokines participating in sepsis immunosuppression (30–34). LPS tolerent macrophage show decresed expression of costimulatory molecules (CD86, *etc.*), decreased expression of MHC-II-like molecules, and elevated CTLA4 expression, resulting in an antigen-presenting deficiency, decreased ability to produce IL-6, TNF-α and IFN-γ, and an increased ability to produce IL-10 and TGF-β, which induces immunosuppression (29, 35–44). In addition, pathogens induce macrophage apoptosis, pyroptosis, necroptosis, and parthanatos that make it impossible for immune cells to proliferate effectively, thus making it difficult for the host to effectively respond to pathogens (33, 45). The above dysregulated macrophage function, including macrophage overactivation, macrophage phenotypic reprogramming, and programmed macrophage death can be regulated by nanomaterials to achieve macrophage-targeted therapy in sepsis.

## The rationality of nanomaterial targeting macrophage

**Figure 1:**
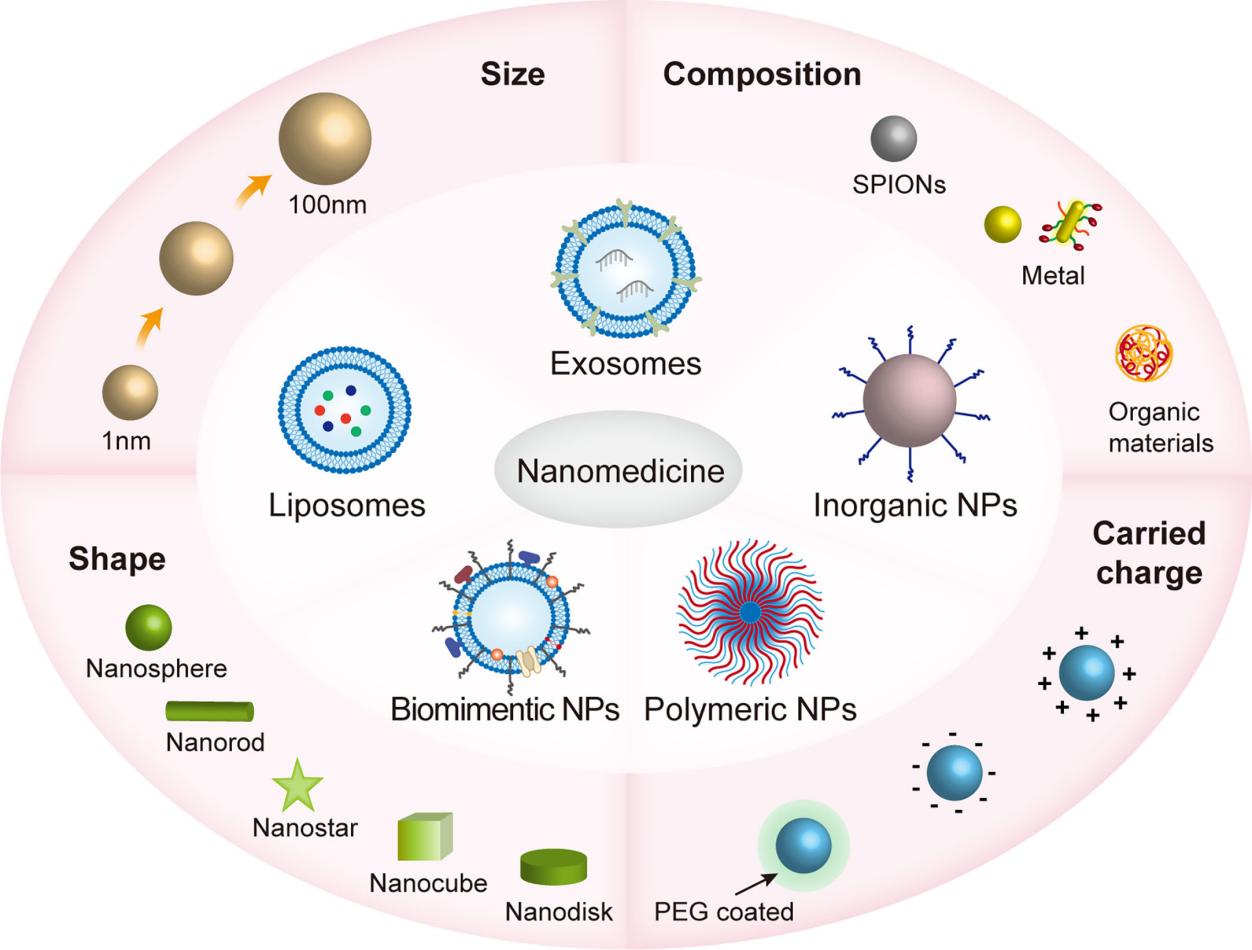
The various properties of nanomaterials in nanomedicines. Nanomaterials are subdivided into polymorphic nanoparticles (NPs), liposomes, biomimetic NPs, inorganic NPs, and cell-derived exosomes, which range in size from approximately 1 to 100 nm and have different shapes, including nanospheres, nanorods, nanostars, nanocubes, nanodisks, etc.; consist of different materials, including SPIONs, metallic fabrics, and organic materials; and are both positively/negatively charged on the surface or coated by PEG. These physicochemical properties influence how efficiently nanomaterials enter the cell and the mode of action that affects cellular activity. The size and shape of the nanomaterial can determine the way it enters the cell, such as clathrin-mediated endocytosis (120nm), and clathrin- and caveolae-independent endocytosis (~60nm) (51).. The surface charge of nanoparticles affects the cell membrane state, for example, the binding of negatively charged nanoparticles to cellular lipid bilayers causes local gelation, while the binding of positively charged nanoparticles to cellular lipid bilayers causes the flow of phospholipid bilayers (52). Polyethylene glycolization reduces premature removal of NPs from the cycle (53).

NPs are synthesized by chemical reduction, wet chemical methods, ligand-mediated self-assembly, electrostatic assembly, polymer encapsulation, and nanoprecipitation and so on (46, 47).Nanomaterials have unique advantages over ordinary drugs, which include tunable properties (e.g., structural size and composition, carried charge and surface chemical properties), surface functions (e.g., target ligands and molecules) and specific binding features (48, 49). The core of nanomaterials is nanoparticles, which are particles of nanoscale size (from 1nm to 100nm) (47). Engineered NPs are classified into polymeric NPs, liposomes, biomimetic NPs, exosomes and metal/inorganic NPs (47, 50). Different material compositions (such as SPIONs, metallic fabrics, and organic materials), surface coatings (such as positively/negatively charged on the surface or coated by PEG) and shapes (such as nanospheres, nanorods, nanostars, nanocubes, nanodisks, etc.) give NPs different properties that affect the efficiency and mode of action of nanomaterials into cells (47, 51) (**Figure 1**).

Polymeric NPs not only protect “antigen” from enzymatic digestion but also have APC targeting, easy surface modification, biodegradable, nontoxic and nonimmunogenic features. Drugs can be loaded on the surface or inside polymeric nanospheres and nanocapsules to avoid enzymatic digestion while crossing biological barriers to the target region (54). Liposomes compose phospholipids and cholesterol and can encapsulate both lipid-soluble and water-soluble drugs, preventing the drug from rapidly degrading and reducing adverse reactions by preventing direct contact with the systemic circulation (52, 53). Based on the properties of receptor-ligand binding, target cell-specifical ligands can be assembled on the surface of liposomes to facilitate receptor-mediated liposome endocytosis and promote the entry of liposomes and their loaded drugs into target cells (52, 53). Biomimetic NPs are NPs formed by attaching natural ligands or functional components, such as cell membranes, to the surface of engineered NPs (16). Cell membrane coating nanotechnology has been developed to synthesize biomimetic NPs by covering the surface of synthesized NPs with cell membranes prepared using techniques such as osmotic pressure difference, chemical interference, electroporation, and ultrasonic treatment (55). Macrophage membrane-coated biomimetic NPs combine the unique biochemical functions of macrophages that can achieve targeted drug delivery with low immunogenicity (16, 56). Exosomes defined as a type of extracellular vehicles between 30 and 150 nm can transfer the encapsulated biomolecules (such as DNA, RNA, proteins, lipids and metabolites) from the donor cell to the recipient cell, thus triggering cell phenotypic changes participating in a variety of immune responses (57–63). Exosomes possess the advantages of inherent stability, high delivery efficiency, and ability to cross biological barriers (62, 64–66). Inorganic nanomaterials have superior optical and magnetic characteristics and a high surface-area-to-volume ratio, thus making them ideal for molecular detection, drug delivery, immunomodulation, *etc.* For example, nanoscale noble metals (*e.g.*, AuNPs) exposed to light exhibit localized surface plasmon resonance (LSPR) phenomena, resulting in the improved sensitivity of molecular detection (67, 68). Moreover, cerium nanoparticles significantly attenuated the total superoxide flux in macrophages (69).

**Figure 2:**
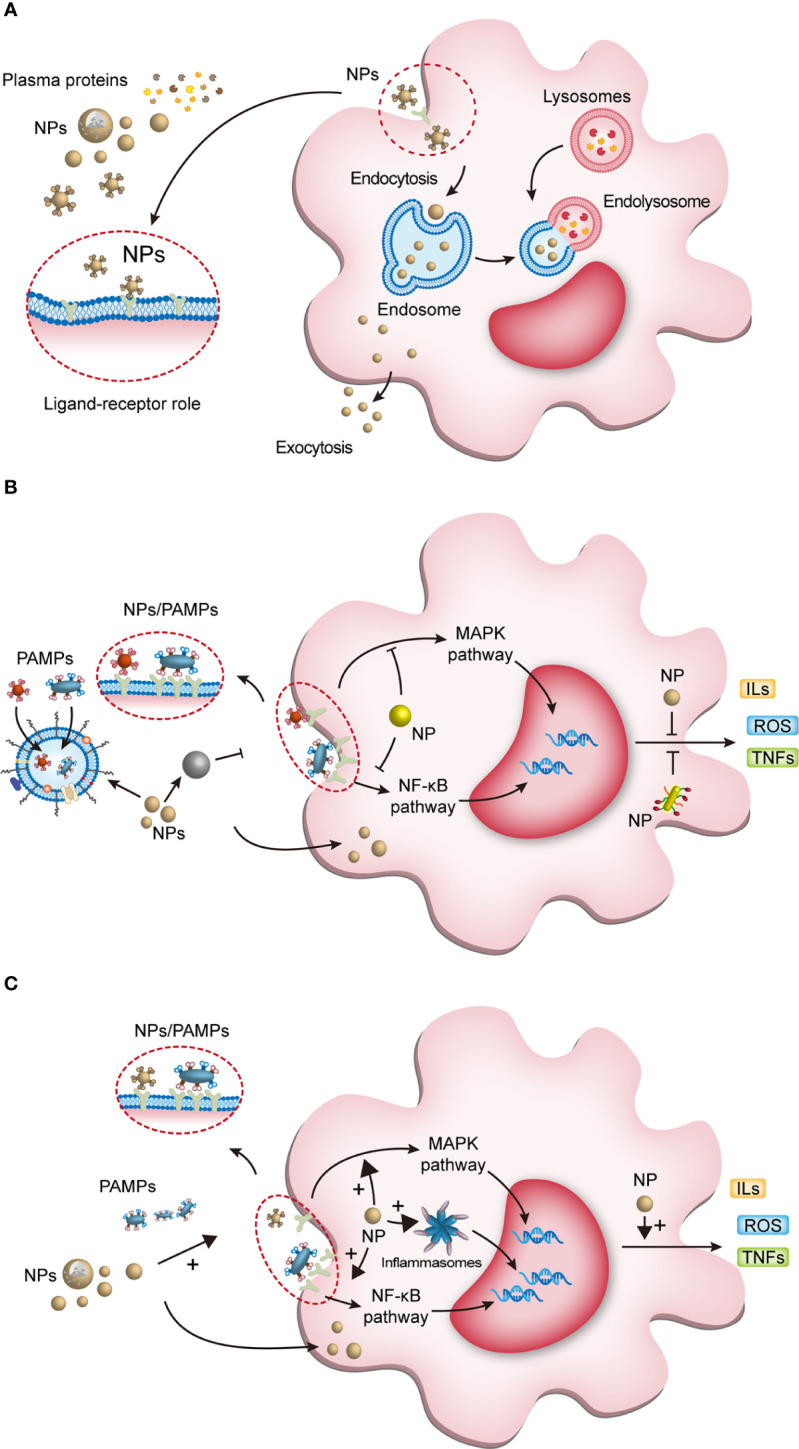
The interaction of NPs with macrophages and NPs modulating macrophage anti/pro-inflammatory function. **(A)** Once NPs enter the body, they bind to plasma proteins and enter macrophages. Some endosomes containing NPs degrade the processed NPs and release them extracellularly to exert active effects; the other endosomes fuse with lysosomes to form endolysosomes, which exert intracellular effects. **(B)** First, NPs can eliminate macrophage activation by phagocytosis and confinement of pathogen-associated molecular pattern molecules (PAMPs); second, they inhibit PAMPs interacting with pattern recognition receptors (PRRs); third, NPs entering the cytoplasm inhibit the transduction of inflammatory signaling pathways; and finally, NPs inhibit the release of active products of inflammatory pathways and control the cell and tissue damage caused by overactivated macrophages. **(C)** NPs modulate macrophage proinflammatory activity. NPs can enhance PRR activation to initiate macrophage inflammation. After entering the cytoplasm, NPs activate downstream pathways and inflammasomes to induce proinflammatory factor production. NPs, nanoparticles; PAMPs, pathogen-associated molecular pattern molecules; ROS, reactive oxygen species; ILs, interleukins; TNFs, tumor necrosis factors; MAPK, mitogen-activated protein kinase; NF-κB, nuclear factor-kappa B.

Through nanoprecipitation, emulsion polymerization, electroporation, film dispersion, ultrasonic dispersion, reverse-phase evaporation method, *etc.*, scientists can program the physicochemical properties of NPs. Engineered NPs are both for loading drugs targeting macrophages to improve their bioavailability and for modifying the structure of nanomaterials to modulate macrophage function by supramolecular chemistry. NPs exerting their intracellular or extracellular biological activities after being recognized and endocytosed by macrophages (**Figure 2**) (46, 47, 55, 70). Plasma contains various proteins, which can bind to the nanoparticles (NPs), and once NPs enter the host and contact with plasma proteins, they acquire a new biological characteristic called protein corona (PC) (71). PC changes the physicochemical properties of NPs, including surface charge, size, aggregation state and antigenic epitopes; and these changes preferentially in turn affect the pharmacokinetics, biodistribution and therapeutic effects of NPs (71). Binding to certain proteins lead to recognition of NPs by the mononuclear phagocyte system (MPS) (72). For example, IgG, fibrinogen and complement proteins, promote the uptake of NPs by the MPS utilizing the corresponding receptors expressed on the phagocyte surface, such as scavenger receptor on Kupffer cell. In addition, IgG and complement C3b promote the uptake of NPs by monocytes (73).

**Figure 3:**
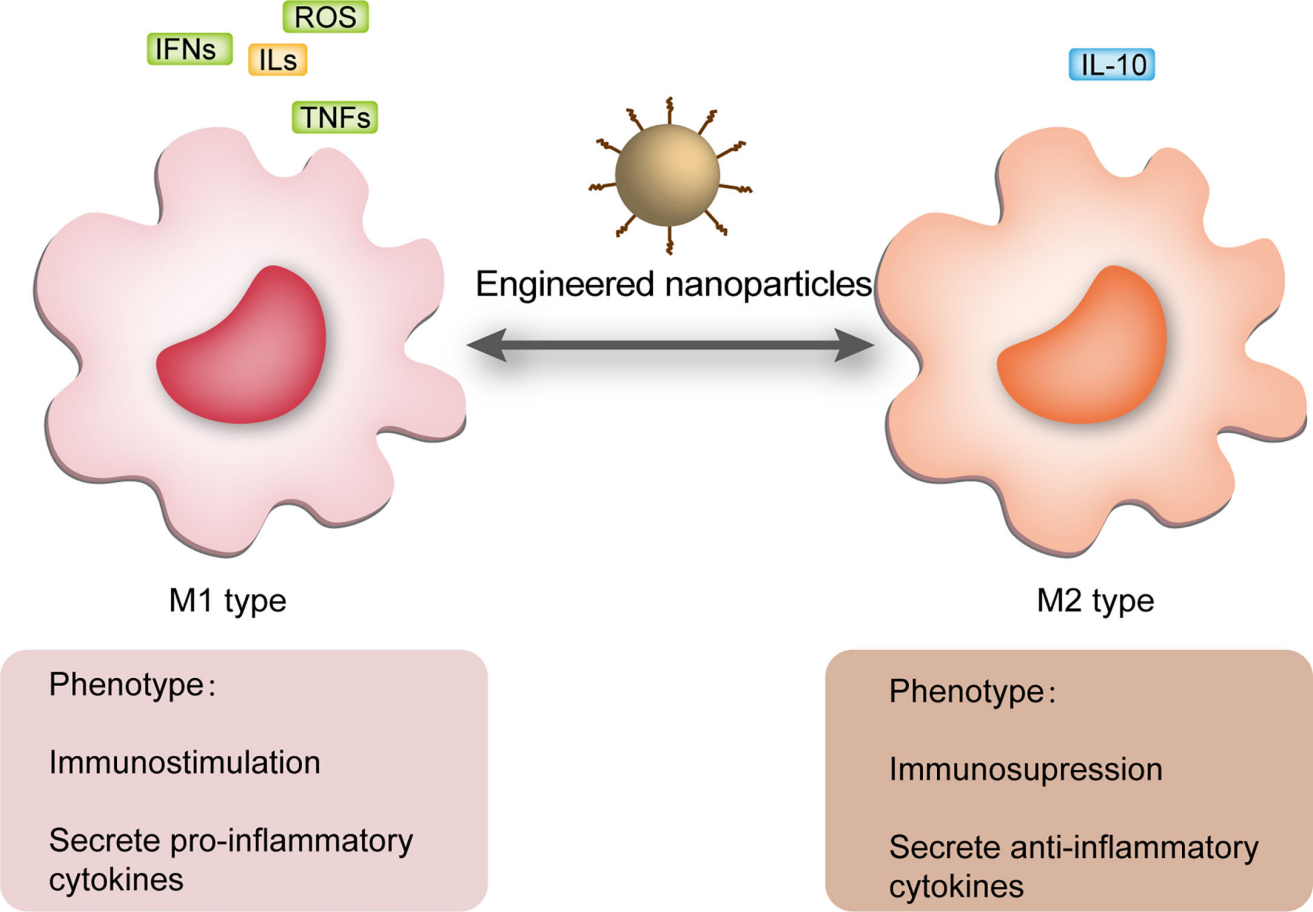
NPs regulated macrophage polarization. M1 macrophages usually act in the sepsis cytokine storm state and release a large number of pro-inflammatory mediators, including ROS, IFNs, ILs and TNFs, while M2 macrophages usually appear in the immune paralysis phase and secrete anti-inflammatory mediators, the most characteristic of which is IL-10. During periods of inflammatory overstimulation, sustained release of pro-inflammatory mediators from M1 induces damage to the organism, while massive activation of M2 macrophages during periods of immune paralysis increases the risk of secondary infection. NPs can regulate macrophage polarization in different periods of sepsis improving its prognosis. NPs, nanoparticles; ROS, reactive oxygen species; ILs, interleukins; IFNs, interferons; TNFs, tumor necrosis factors.

**Figure 4:**
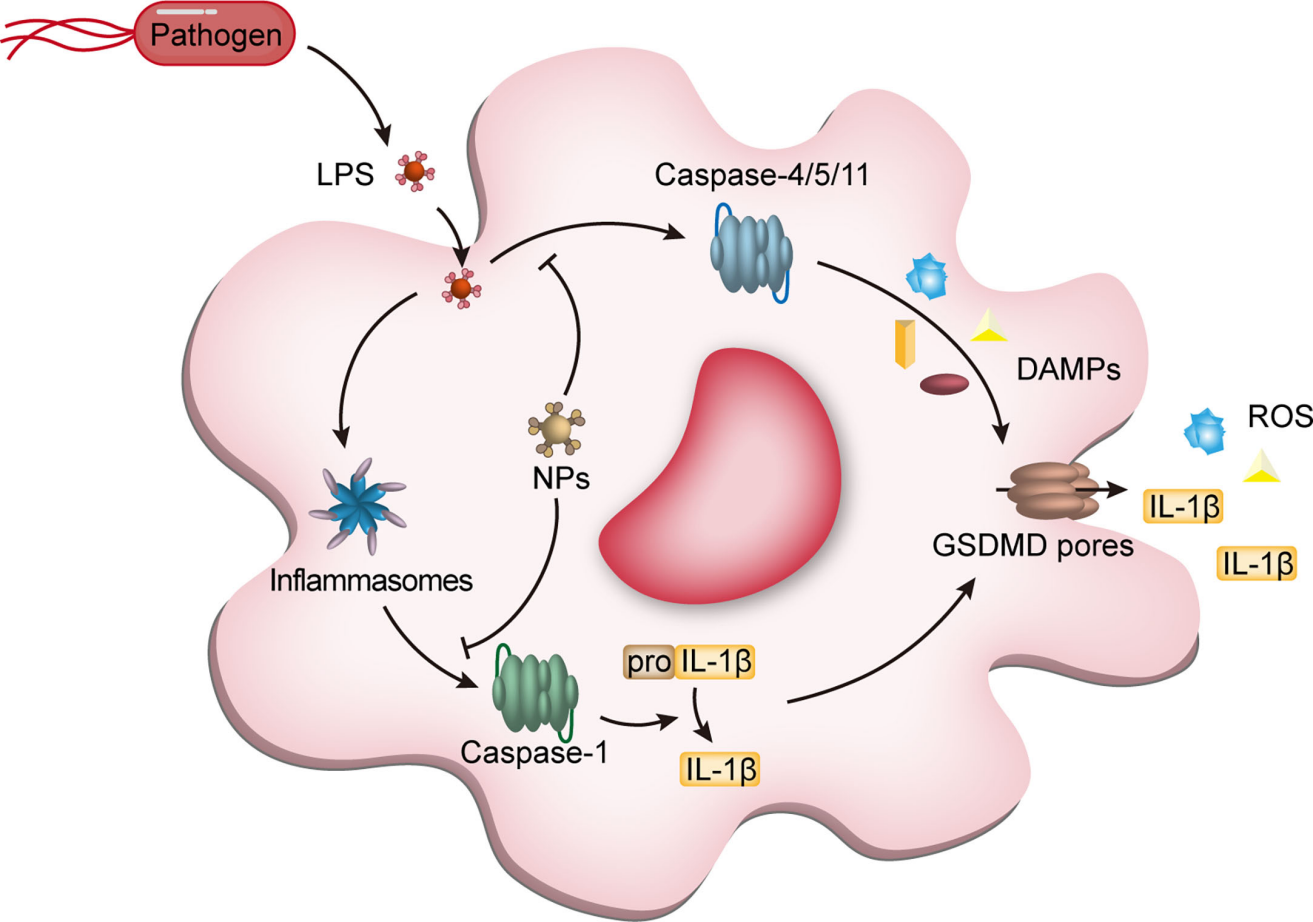
NPs interfere with macrophage pyroptosis. Pyroptosis, a class of programmed cell death dependent on pro-inflammatory caspases (caspase-1,-4,-5 from humans and caspase-1 and -11 from mice) and gasdermin D (GSDMD), is an important type of programmed macrophage death. After recognition of LPS derived from pathogens by macrophage intracellular receptors, activation of caspases triggers the cleavage of GSDMD and IL-1β, which accumulates in the cell membrane to form pores causing cell membrane collapse, accompanied by the release of inflammatory cytokines, including IL-1β, ultimately leading to cell death. The NPs entering the cells can block the activation of caspase-1 and caspase4/5/11, reducing the release of DAMPs and avoiding unnecessary tissue and cell damage. NPs, nanoparticles; LPS, lipopolysaccharides; IL-1β, interleukin-1β; DAMPs, damage-associated molecular pattern molecules; GSDMD, gasdermin D.

**Figure 5:**
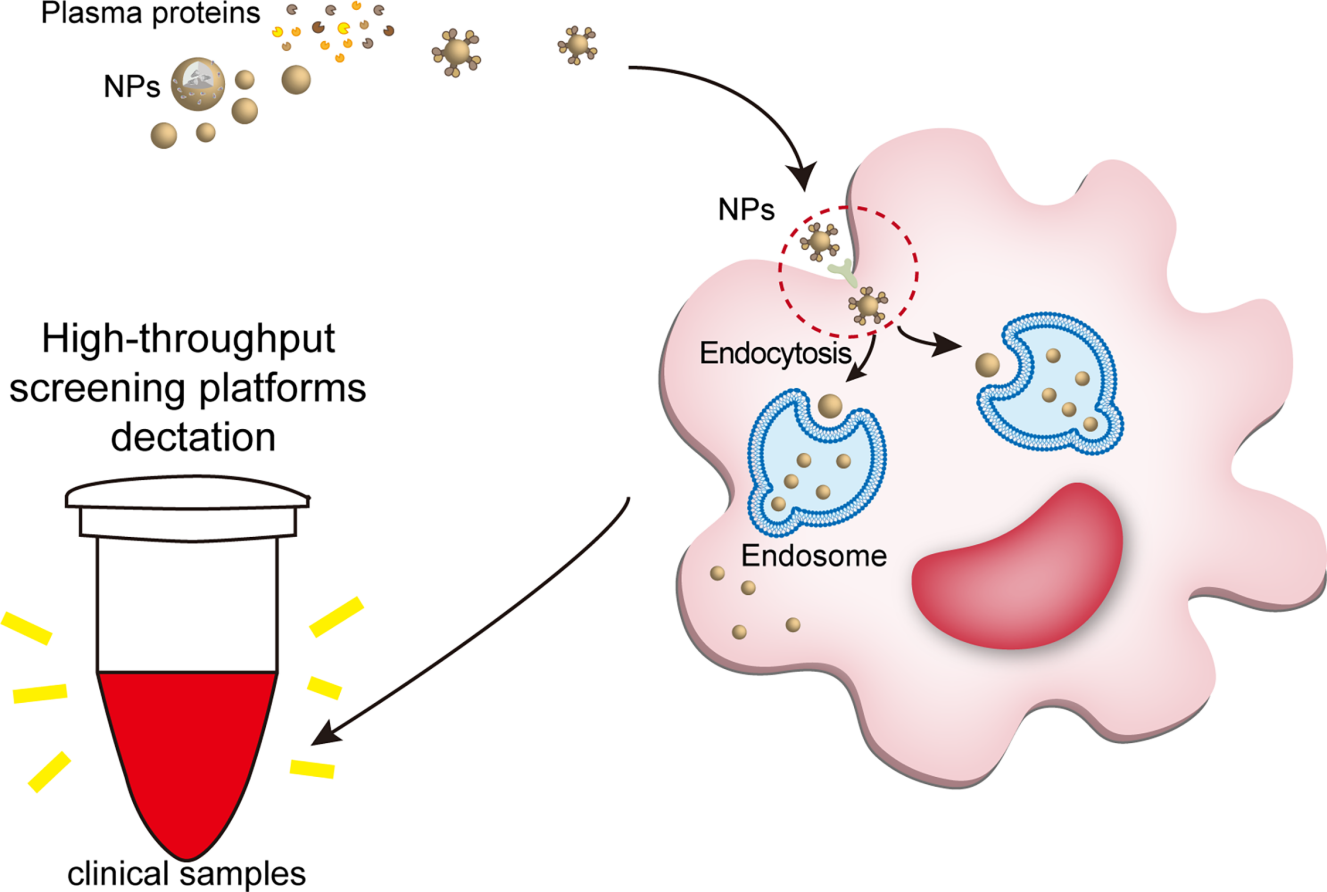
NPs engulfed by macrophages can be detected by clinical tools. The large number of proteins bound to the NP can trigger immediate recognition by macrophages. Ligand-receptor role is a part of way that plasma protein helps NPs enter macrophages. When nanomaterials interact with biological fluids, after the formation of a protein corona, their main requirement is to interact with cell membranes (especially the molecule in the cell membranes surface) to show their biological effects, such as ligand-receptor role. For example, IgG, fibrinogen and complement proteins, promote the uptake of NPs by the macrophages utilizing the corresponding receptors expressed on the phagocyte surface, such as scavenger receptor on Kupffer cell. Once the NPs are endocytosed into macrophages, the nanotracer in macrophages of the samples can be tested by high-throughput screening platforms, such as optical nanosensor and other nano-detector. NPs, nanoparticles.

Nanomaterials are successfully used in diverse diseases (**Table 1**), especially sepsis (**Table 2**) (56, 85, 116). Nanomaterials targeting macrophages mainly lie in the therapeutic aspect, including as drug carrier or nanodrug to regulate macrophage anti-inflammatory/pro-inflammatory function (**Figure 2**), macrophage reprogramming (**Figure 3**) and programmed macrophage death (**Figure 4**) (16, 22, 112, 113, 115, 117). In the regulation of macrophage anti-inflammatory/pro-inflammatory function, PAMPs and pattern recognition receptor (PRR) shows great potential (35). The process of recognition and phagocytosis of nanomaterials by macrophages approximates the mutual recognition of PAMPs and PRRs (35). NP delivery platforms in combination with PAMPs or their synthetic mimics hold great promise in immunomodulatory therapy using synthetic or natural polymers, lipid-polymer hybrids and self-assembled compounds to constitute nanodelivery systems that capture or adsorb TLR ligands and modulate innate immune responses (118, 119). CpG sequences are typical PAMPs, which when bound to PLG can be widely recognized and phagocytosed by antigen-presenting cells (APCs), including macrophages, to enhance the host immune response (120). TLR receptors of dendritic cells and monocytes have been shown to recognize alginate-coated chitosan nanogels, affecting the TLR ligands Pam3Cys-SK4 or CpG-ODN involved in the regulation of their immune function, inducing the release of IL1-β, IL-6, TNF-α, and IFN-α (121). After enter the macrophages, NPs which load immunomodulatory drugs can promote/inhibit NK-κB/MAPK pathway to modulate macrophage function (84, 122).What’s more, PRRs assemble into inflammasomes after detecting pathogenic microorganisms or DAMPs in the cytoplasmic matrix of the host cell (123). In macrophages, PRRs assemble into inflammasomes upon detection of pathogenic microorganisms and danger signals in the cytoplasmic matrix of host cells. Silica nanoparticles (SiO (2) NPs) Silica enters the cell and generates ROS, which activate the inflammasome, including caspase-1, ASC multimerization, and promote IL-1β and IL-18 expression in macrophages (124, 125). Multi-walled carbon nanotubes (MWCNTs) and asbestos induce NLPR3 inflammasome activation in macrophages, and this activation depends on reactive oxygen species (ROS) production, histone B activity, P2X7 receptors, and Src and Syk tyrosine kinases (126). In addition, the specific deposition of imaging agents in macrophages can be detected with the aid of an imaging device, enabling time- and space-specific monitoring of macrophages (**Figure 5**).

**Table 1:** The classification and mechanism of nanoparticles targeting macrophages.

Nanoparticles	Techniques	Mechanisms	Principles/methods	Model/cell lines	References
AuNPs	Functionalized with para-mercaptobenzoic acid (p-MBA)	Detection of macrophages phagolysosomal pH	Surface-enhanced Raman spectroscopy	Peripheral blood mononuclear cells isolated from whole blood of human subjects	(74)
Liposomes	Synthesized with NO probes *via* thin film hydration approach	Detection of nitric oxide release by activated M1 macrophages	Near-infrared (NIR) light sensing nitric oxide probes	RAW264.7 macrophages	(75)
Cellulose nanocrystals	Linked to PEGylated biotin and perylene diimide (PDI)-based near-infrared organic dye	Labelling, imaging, and long-time tracking for macrophages	Ultraviolet–visible absorption spectroscopy and fluorescence emission spectroscopy	J774A.1 macrophages	(76)
Liposomes	Modified by matrix metalloproteinase-2 (MMP-2) responsive peptide (peptide E5) *via* the film dispersion method	Drug-delivery systems	Ultraviolet–visible spectrophotometry and reversed-phase high-performance liquid chromatography (RP-HPLC) analysis	C57BL/6 male mice	(77)
Liposomes	PEGylated liposomes containing IFN-γ	Drug-delivery systems	Nitric oxide test and biodistribution work	J774A.1 macrophages	(78)
Polymeric NPs and AuNPs	Coated with collagen in the first layer and subsequently modified with biotin-quat188-chitosan in the outer layer *via* Layer-by-Layer (LbL) assembly technique	Drug-delivery systems	Fourier transformed infrared spectroscopy	RAW264.7 macrophages	(79)
Biomimetic NPs	Macrophage membrane coating on the surface of active Ingredients	Drug-delivery systems	The specific targeting of macrophage membrane to lesions	RAW264.7 macrophages/C57BL/6 mice	(56, 80, 81)
Multiwalled carbon nanotubes		Macrophage activation and enhance phagocytosis	Tim4 recognizes MWCNTs through aromatic interactions and mediates phagocytosis/NLRP3 inflammasome activation	C57BL/6J mice	(82, 83)
Poly (3-hydroxybutyric acid-co-hydroxyvaleric acid) (PHBV) NPs	Developed by water-oil-water double emulsion method	Macrophage activation and enhance phagocytosis	Continuously activate NOD1	RAW264.7 macrophapes	(84)
Biomimetic NPs	Cell-membrane-coating nanotechnology	Macrophage activation and enhance phagocytosis	Elicit macrophage immune r esponses *via* CD47 and SIRPα	B16F10 mice	(85)
PLGA, silica NPs	Nuclear magnetic resonance-based metabolomics	Induce proinflammatory factors production	Induce TNF-α production.	RAW 264.7 macrophages	(86)
Carbon dots	Produced by microwave-assisted pyrolysis of organic precursors	Induce proinflammatory factors production	Induce NLRP3 inflammasome activation and IL-1β, IL-8 release	BALB/c mice	(87)
Polymeric NPs		Induce proinflammatory factors production	Induce ROS production	NR8383 rat macrophages/RAW 264.7 macrophages	(88, 89)
Silica and superparamagnetic iron oxide (SPION) NPs	Prepared by chemical coprecipitation	Inhibit macrophage phagocytosis	Diminish phagocytic activity of macrophage toward S. pneumoniae	Bone marrow-derived macrophages isolated from C57/BL6 mice	(90)
Methyl palmitate NPs	Produced by combination natural fatty acid methyl palmitate with albumin	Inhibit macrophage phagocytosis	Induce macrophages into a transient and reversible state of dormancy	C57BL/6J mice	(91)
Polymeric NPs	NF-κB/p65 antisense oligonucleotide loaded chitosan	Inhibit proinflammatory pathways	Inhibit NF-κB/p65 pathway	RAW 264.7 macrophages	(92)
Au NPs	Combined with ginsenoside compound K (CK) and peptide CopA3	Inhibit proinflammatory pathways	Inhibit NF-κB and MAPK pathways	RAW 264.7 macrophages	(93)
Lipoaspirate NPs	Incorporated with guanabenz	Inhibit proinflammatory pathways	Inhibit TLR4 pathway	Raw264.7 macrophages	(94)
Drug-free amphiphilic NPs	Generated by self-assembly of hydrolyzed galactomannan (hGM)-linked copolymers	Promoting M1 to M2 macrophage polarization		Raw264.7 macrophages	(95)
Liposomes	Conjugated with protein G	Promoting M1 to M2 macrophage polarization	Showed by reduced IL-1α, IL-6, and TNF-α production and increased IL-10 production	C57/BL6 mice and Raw264.7 macrophages	(96)
Selenium-based layer-by-layer nanocomplexes	Combined with polyethyleneimine	Promoting M1 to M2 macrophage polarization	Evidenced by decrease in NOS-2 and TNF-α mRNA expression	Raw264.7 macrophages	(97)
Lanthanide upconversion NPs	Near-infrared light -controlled cyanobacteria micronanodevice	Promoting M1 to M2 macrophage polarization	Reduce HIF-1α expression	C57/BL6 mice and BALB/c mice	(98)
Biomimetic Au NPs		Promoting M2 to M1 macrophage polarization	Induce proinflammatory cytokine, ROS production and glutathione consumption	BALB/c mice	(99)
Micellar nanostructure of supramolecule	Accompanied with efficient cytoplasmic translocation and tunable photoconversion	Promoting M2 to M1 macrophage polarization	Induce of apoptotic proteins and inhibit metastasis-associated proteins	4T1-tumor-bearing mice	(100)
AuNPs	Functionalized with mangiferin	Promoting M2 to M1 macrophage polarization	Evidenced by enhanced IL-12 and TNF-α, and reduced IL-10 and IL-6	RAW 264.7 macrophages	(101)
Nano enzyme and PEGylated iron manganese silicate NPs		Promoting M2 to M1 macrophage polarization	Exhibit peroxidase-like and catalase-like activities to decompose hydrogen peroxide (H2O2) into hydroxyl radicals (-OH) and oxygen (O2)	CT26-tumor-bearing mice	(102)
NPs encapsulated by macrophage exosomes	Combination of PLGA-based NPs with exosome membrane from macrophages	Specific target to macrophages in lesions	Regulate macrophage phagocytosis and macrophage polarization	C57BL/6 mice	(103, 104)

**Table 2:** The classification and mechanism of nanoparticles targeting macrophages in sepsis.

Nanoparticles	Techniques	Mechanisms	Principles	Model/cell lines	References
Dextran NPs	64Cu-Macrin positron emission tomography (PET) imaging	Nanotracer for macrophage	Quantitative noninvasive assessment for spatiotemporal macrophage dynamics	Cecal ligation and puncture (CLP) -induced C57BL/6J sepsis mice	(105)
Superparamagnetic iron-oxide NPs	Quantitative susceptibility mapping magnetic resonance (QSM-MRI)	Monitoring tools based on macrophage phagocytosis	Quantitative susceptibility mapping magnetic resonance for NP phagocytosis by macrophages	Lipopolysaccharide (LPS)-treated RAW 264.7 macrophages	(106)
Liposomes	Constructed by antimicrobial peptide- cathepsin B Mrna and vitamin	Drug-delivery system targeted for macrophages	Promote the accumulation of NPs in macrophage lysosomes to kill multidrug-resistant bacteria	RAW 264.7 macrophages and multidrug-resistant bacteria -induced sepsis C57BL/6 mice	(107)
Monophosphoryl Lipid A (MPLA)@PLGA NPs	Contain a NOD2 agonist, TLR4 agonist and alginate (ALG)	Macrophage activation and phagocytosis	Enhance the phagocytic and bactericidal function of macrophages.	Raw 264.7 macrophages and CLP-induced sepsis C57BL/6 mice	(24)
Biomimic macrophage NPs	Contain a recyclable polymeric NP covered with macrophages membrane have similar antigenic external of macrophages	Macrophage activation and phagocytosis	Capture and eliminate LPS and inflammatory factors	LPS-induced sepsis BALB/c mice	(23)
Cerium oxide NPs		Induce antioxidant and anti-inflammatory activity	Reduce the superoxide flux of mitochondrial electron transport chain (METC) and plasma membrane NADPH oxidase (NOX), and downregulate proinflammatory cytokines release	LPS-induced sepsis Sprague Dawley rats	(26, 69, 108)
Metal and polymeric NPs	a mannosylated disulfide cross-linked polyethylenimine (ssPEI) (mSP)-coated bovine serum albumin-reduced MnO2 (M SPAM) nanoassembly	Induce antioxidant and anti-inflammatory activity	Decompose toxic H2O2 to oxygen and water, prevent proinflammatory cytokines secretion	LPS-induced sepsis C57BL/6 mice	(109)
Cerium oxide NPs		Target inflammatory pathways.	Reduce MAPK/NF-κB mediated pathways activation	RAW264.7 cells and CLP-induced sepsis Sprague Dawley rats	(25, 26, 69)
Poly(Lactic Acid) iNPs	Prepared by oil-in-water (o/w) emulsion-solvent evaporation (SE) technique	Target inflammatory pathways.	Elimination of NF-κB p65 and MAPK p38 activation	RAW264.7 cells and LPS-induced sepsis C57BL/6 mice	(110, 111)
Au NPs		Mediate macrophage polarization	Demonstrated by the lower supernatant TNF-α and IL-1β and higher Arginase 1	CLP-induced sepsis ICR mice	(112)
SPION of γ-Fe2O3 NPs		Mediate macrophage polarization	Induce TRAF1-dependent polarization	LPS-and CLP-induced C57BL/6	(113)
SPIONs of γ-Fe2O3 NPs		Regulated cell death	Induce Cav1-Notch1/HES1-mediated autophagy	RAW264.7 cells and LPS-induced sepsis C57BL/6 mice	(114)
Lactoferrin NPs	Loaded with disulfiram	Regulated cell death	Inhibit GSDMD-induced pyroptosis	C57BL/6 and BALB/c mice	(115)

## Nano drug carriers targeting macrophage

Drugs can be encapsulated and sequestered by NPs or covalently attached to the surface of NPs, enhancing drug-targeted delivery and release and/or improving drug biodistribution and/or bioavailability to modulate the anti-inflammatory/pro-inflammatory activity of macrophages, macrophage reprogramming and macrophage pyroptosis (17). In addition, the release time and site of the modified nanodrug can be controlled after being triggered by environmental physicochemical properties (*e.g.*, pH and enzyme action), thus regulating the eff ects of the drug in plasma and cells (49, 127).

### Polymeric NPs as drug carriers target macrophages

Polymeric NPs can facilitate targeted drug delivery due to easy surface modification, biodegradable, nontoxic and nonimmunogenic features (128–130).

Polymeric NPs promote macrophage anti-inflammatory activity in sepsis overactivation stage. Chitosan, as a kind of polymeric NPs, improves drug delivery efficiency and controlled release (22). For example, Hongsa et al. designed a modified biotin-quat 188-chitosan (Bi-QCS) and collagen nanodrug carrier (Bi-QCS-AuNPS@collagen) wrapped in AuNPs surface (79). Bi-QCS significantly improved the uptake of loaded drugs by macrophages, and chitosan improves physicochemical stability, controls drug release and promotes its anti-inflammatory activity (79). Compared with conventional AuNPs, Bi-QCS-AuNPs@collagen has higher drug loading and promotes apparent anti-inflammatory role in RAW264.7 macrophage (79). In addition, reactive oxygen species (ROS) and pH-sensitive polymeric chitosan/alginate hydrogel NPs loaded with curcumin effectively avoid the hydrolysis of digestive fluid and directly target macrophages to exert anti-inflammatory effects *via* TLR4-MAPK/NF-κB pathway inhibition; among them, chondroitin sulfate promotes macrophage targeting of NPs, while chitosan/alginate hydrogel protects NPs from being destroyed by digestive juices (122). A nanocomposite synthesized by chitosan and antimicrobial peptides (AMPs) significantly inhibited NF-κB/MAPK pathway activation by LPS in RAW264.7 macrophages as well (131). Moreover, chitosan was developed to carry NF-κB/p65 antisense oligonucleotides that target macrophages to inhibit NF-κB/p65 signaling and downstream release levels of inflammatory factors such as IL-1, IL-6 and TNF-, in LPS-stimulated RAW264.7 macrophages (92). Rajendrakumar et al. developed a mannosylated disulfide cross-linked polyethyleneimine (ssPEI) (Msp)-encapsulated bovine serum albumin-reduced manganese dioxide (MSPAM) nanocomplex that effectively avoided organ damage caused by macrophages in a sepsis model (109). Hydrophilic bovine serum albumin-reduced manganese dioxide (BM) NPs self-assembled with cationic mannose cross-linked polyethyleneimine (Msp) from MSPAM nanocomplexes scavenged H2O2, inhibited HIF1α expression and reduced serum TNF-α and IL-6 (109).

Polymeric NPs promote macrophage pro-inflammatory activity in sepsis immunosuppression stage. Apoptosis, endotoxin tolerance, metabolic reprogramming, and changes in inflammatory pathway are involved in the immunosuppressive state of sepsis (37). In the immunosuppression stage, the host often dies due to organ dysfunction (132), and nanomaterials can induce macrophage proinflammatory reaction to improve the survival rate of patients (95). iE-DAP is a drug that promotes intracellular receptor NOD1 activation and induces pro-inflammatory factor gene expression, but cannot be internalized by macrophage. After be encapsulated by poly(3-hydroxybutyrate-co-3-hydroxyvalerate) (PHBV), iE-DAP-PHBV can be effectively internalized into macrophages activateing NOD1 signaling to induce activation of the NF-κB pathway and secrete IL-6 and TNF-α against inflammation (84). Zhao et al. loaded monphosphatidyl lipid A (MPLA) and muramyl dipeptide (MDP) into poly(lactide-co-glycolide) (PLGA) NPs and combined them with alginate (ALG) to develop two-phase release immunostimulatory composite NPs (MDP+P-M@ALG). MDP+P-M@ALG improves macrophage phagocytic and bactericidal functions, the survival of CLP-induced sepsis mouse models and the resistance of surviving mice to secondary infections, providing long-term sepsis protection (24).

Polymeric NPs regulate macrophage reprogramming. Macrophages activated in inflammation are generally divided into two types, pro-inflammatory M1 and anti-inflammatory M2 macrophages (133). NP can change the inflammatory environment by regulating the activated macrophage state and thus treat diseases (77). For example, Jiang et al. prepared chitosan-based nanoparticles (CN) loaded with tripolyphosphate that dynamically regulated M1-M2 macrophage reprogramming. In M1-like macrophages, CN decreased CD86 and iNOS expression, and increased Arg-1 and IL-10 expression; in M2-like macrophages, CN decreased Arg-1 expression, and increased CD86, iNOS and TNF-α expression. The biphasic polarization was achieved by STAT-1/STAT-6 signaling pathway transformation. Therefore, CN alter macrophage polarization homeostasis and thus can be used for treating sepsis (134).

Polymeric NPs inhibit macrophage pyroptosis. Pyroptosis is a caspases-mediated cell death, which GSDMD accumulates in cell membrane to form pores causing cell membrane collapse, inducing the release of lots of cytokines, including IL-1β, ultimately leading to dramatically abnormal activation of immune cells (135). NPs can serve as carriers prevent its occurrence (115). For example, Ou et al. prepared a disulfiram-lactoferrin nanocomplex (DSF-LF NPs), a naturally occurring powerful antibacterial and anti-inflammatory protein, with DSF, a drug that inhibits gasdermin D (GSDMD)-induced pyroptosis (115). LF binds specifically to low-density lipoprotein receptor-related protein-associated protein (LRP-1) and promotes phagocytosis of NPs by macrophages, and has immunomodulatory effects (115). Utilizing the immunosuppressive activity of LF and DSF, DSF-LF NPs effectively inhibit macrophage pyroptosis and proinflammatory cytokine release process with significant efficacy in LPS-induced sepsis (115). In addition, a siHMGB1 liponanocomplex can be engulfed by macrophage *via the* mannose receptor to form endolysosomes. Endolysosomes can release active factors to silence the transcription of high mobility group box protein 1 (HMGB1), thus inhibiting pyroptosis (136).

### Liposomes as drug carriers target macrophages

Liposomes are widely used as drug carriers for small molecule, peptide, protein, gene and antibody delivery due to their high drug encapsulation, low drug toxicity, good targeting, good biocompatibility, biodegradability, and optimized biodegradability pharmacokinetic properties (77, 137–139). Liposomes are phagocytosed by macrophages after entering the body through intravenous injection, forming a natural aggregation effect and realizing macrophage targeting (140).

Liposomes promote macrophage anti-inflammatory activity in sepsis overactivation stage. Liposomes loaded guanabenz regulates macrophage anti-inflammatory activity through eukaryotic initiation factor 2 (eIF2α) dependent signaling, which downregulates IL-6 and cyclooxygenase 2 (COX-2) and also through eIF2α non-dependent signaling, which downregulates IL1β, TNFα, significantly reduced the cytokines secreted by macrophages (94).

Liposomes promote macrophage pro-inflammatory activity in sepsis immunosuppression stage. Hou et al. constructed an antimicrobial peptide, cathepsin B mRNA (AMP-cat B mRNA), encoding AMP-IB367 and Cat-B, which was encapsulated in vitamin liposomes. The vitamin liposomes promote the accumulation of NPs in the lysosomes of macrophages (107). Such macrophages assembled with AMP-cat-B@VLMP could eliminate MDR bacteria in septic mice in an immunosuppressive state (107), providing an alternative strategy to overcome sepsis caused by multidrug-resistant bacteria. Moreover, wheat germ agglutinin (WGA)-modified liposomes encapsulating clarithromycin is used for bacterial target delivery and enhancement of host immune defense by improving the uptake of bacteria by macrophages and inhibiting bacteria growth (141).

Liposomes regulate macrophage reprogramming. M2 macrophages treated with PEGylated liposomes containing IFN-γ expressed elevated NO and decreased arginase levels, suggesting that such liposomes enhanced the targeted delivery of drugs to macrophages and promoted M2 to M1 polarization (78).

### Biomimetic macrophage NPs

Biomimetic macrophage membrane-coated NPs can cross biological barriers, enable the cargo to precisely target the lesion and avoid immune recognition (80, 142, 143).

Biomimetic NPs can both inhibit and promote macrophage phagocytosis. Wang et al. reported a biomimetic NP (MM/RAPNPs) that coats macrophage membranes on the surface of PLGA NPs assembled with rapamycin (RAPNPs) (56). Due to the MM coating, MM/RAPNPs, possessing good biocompatibility, biosafety, and targeting properties, effectively inhibited macrophage phagocytosis *in vitro* (144) and efficiently targeted aggregation to lesions *in vivo* (56). CD47, a ligand for signal-regulated protein-α (SIRPα) on macrophages (145), upon binding to SIRPα, SIRPα activates phosphatase-1 (SHP-1), which contains the Src homology 2 domain, to regulate intracellular signaling and inhibit cellular phagocytosis (146). Related studies reported that magnetic NPs (gCM-MNs) encapsulated by gene-edited cell membranes effectively blocked the CD47-SIRPα signaling pathway and could elicit robust macrophage phagocytosis (85).

Biomimetic NPs promote macrophage anti-inflammatory activity in sepsis overactivation stage. Lu et al. developed a biomimetic nanomedicine (MM-CEP/NLCs) containing cefadroxil (CEP) nanolipid carriers (NLCs) inside and MM encapsulated outside. Due to its biocompatibility and targeting, biomimetic macrophage membrane allows effective accumulation of MM-CEP/NLCs in lung inflammation, achieving sustained drug release and circulation and therapeutic lung inflammation effects (81).

Biomimetic NPs eliminate PAMPs. Macrophage-mimetic NPs (MΦ-NPs) combine polymeric cores with macrophage cell membranes, possessing LPS binding sites (*e.g.*, CD126, CD14 and TLR4) with long circulation times and low toxicity (147, 148). MΦ-NPs can capture and eliminate LPS and damage-associated molecular pattern molecules (DAMPs), reducing the free LPS level in the serum and overexcitation of immune cells and alleviating LPS-induced sepsis in mice (23, 147, 148).

Biomimetic NPs regulate macrophage reprogramming. Engineered macrophages carrying nanodrugs containing oxaliplatin prodrug and photosensitizer induce conversion of M2 macrophages to M1 macrophages as evidenced by increasing of iNOS (M1 marker) and decreasing of Arg-1 (M2 marker), which realized by macrophage-mimetic NP-mediated, light-triggered accurate delivery of drugs (80).

### Exosomes derived from macrophages serve as drug carriers

Exosomes exhibit low immunogenicity, excellent biocompatibility, and immune inertness, and can carry various drugs.

Exosomes regulate macrophage reprogramming. Pei et al. designed an EM-PLGA@Dnmt3aos smart silencer by isolating natural exosomes from M2 macrophages and centrifugation encapsulating a PLGA@Dnmt3aos smart silencer (103). Long non-coding RNAs (lncRNAs) were differentially expressed in M1/M2 macrophages (103). Among them, DNA methyltransferase 3A, opposite strand (Dnmt3aos) is a known lncRNA located on the antisense strand of DNA methyltransferase 3A (Dnmt3a), which highly expressed in M2 macrophages and regulates the expression of Dnmt3a. Smart silencers consist of three small interfering RNAs (siRNAs) and three antisense oligonucleotides (ASOs) that play an important role in mediating sequence-specific silencing of a given target gene. When PLGA@Dnmt3aos-smart silencer encapsulated by M2 macrophage-derived exosomal membranes was injected into allergic asthmatic mice, it effectively targeted M2 macrophages in the lungs and significantly inhibited the production of pro-inflammatory cytokines, demonstrating strong permeability, effective drug delivery, robust targeting, high stability and safety of the exosomes (103). Intercellular adhesion molecule 1 (ICAM-1)/lymphocyte function-associated antigen 1 (LFA-1), and vascular cell adhesion molecule 1 (VCAM-1)/very late antigen 4 (VLA-4), specifically bind to each other (149). ICAM-1 and VCAM-1 are only expressed by macrophages activated by LPS, and LFA-1 and VLA-4 are upregulated in exosomes derived from M2 macrophages, thus enabling targeted recognition of M2-derived exosomes with LPS-activated macrophages (150). The use of exosomes derived from M2 macrophages will encapsulate the plasmid DNA encoding IL-10, avoid the degradation of plasmid DNA by nucleases and adverse reactions of plasmid DNA (150). The exosomes realize the targeted transporting to M1 macrophage, and enhance the reprogramming of the M1 type to the M2 type macrophages, which was demonstrated by the upregulation of IL-10 and IL-4 and the downregulation of IL-1β and TNF-α (150).

### Inorganic NPs as drug carriers target macrophages

The tunable optical and electronic properties, simple synthesis techniques, and biocompatibility of carbon nanomaterials make them promising for applications in *in vitro* and *in vivo* biosensing, bioimaging, and drug delivery (151, 152).Metal NPs are representative of inorganic NPs, which offer considerable advantages as therapeutic platforms due to their high drug-carrying capacity, low immunogenicity, and biotunable targeting properties (153).

Inorganic NPs promote macrophage anti-inflammatory activity in sepsis overactivation stage. Currently, scientists combine ginsenoside compound K (CK) and peptide CopA3 on gold NPs (GNP-CK-CopA3) targeting RAW264.7 macrophages to decrease LPS-induced NF-κB/MAPK pathway activation (93). Gold NPs improve CK and CopA3 delivery efficiency (93). Pretreatment of RAW264.7 cells with GNP-CK-CopA3for 1 h followed by stimulation with LPS for 2 h resulted in significant inhibition of protein IκBα and p38 MAP phosphorylation and degradation in macrophages, indicating that GNP-CK-CopA3 inhibits macrophage anti-inflammatory activity (93).

In organic NPs promote macrophage pro-inflammatory activity. Steckiewicz et al. reported that AgNPs loaded with chlorhexidine or metronidazole enhance the antimicrobial roles, and IL-1β expression of RAW264.7 macrophages when compared with conventional chlorhexidine or metronidazole, demonstrating that AgNPs are effective cargo carriers (154).

Inorganic NPs regulate macrophage reprogramming. For example, mangostin-functionalized gold NPs (MGF-AuNPs) were applied to target the NF-kB pathway in splenic macrophages and regulated M2 polarization to M1, which was illustrated by a 10-fold elevation in IL-12, a 50-fold upregulation of TNF-α, and a twofold decrease in IL-6 and IL-10 (101). In sepsis, superparamagnetic iron oxide (SPIO) of γ-Fe_2_O_3_ NPs, which serve as an antibacterial agent, regulated macrophage reprogramming dependent on TRAF1 protein expressed by mesenchymal stem cells to treat septic liver injury (113).

It was concluded that NPs are excellent drug carriers to improve the traditional sepsis therapy efficacy. Equally important, it needs to be emphasized that the functional NPs should be selected properly for sepsis patients in different immune states (such as pro-inflammatory NPs for the immune paralysis state), and the selection of inappropriate NPs will exert adverse effects on the organism (such as anti-inflammatory NPs for the immune paralysis state).

## Nano-molecular drugs targeting macrophage

In addition to being used as drug carriers, nanomaterial itself can be used as macrophage immunomodulator.

NPs inhibit macrophage phagocytosis. As early as 2013, Kodali et al. reported that silica and SPIO NPs could diminish the phagocytic activity of macrophages toward *S. pneumoniae* (90). SPIO have a common recognition receptor with Streptococcus pneumoniae-class a macrophage scavenger receptor (SR-A). SR-A binds SPIO by the charge interaction between the anionic group on the surface of nanoparticles and the lysine rich region of the receptor collagen like (CL) domain. Transcriptional reprogramming induced by SPIO leads to decreased phagocytosis of pathogens by macrophages. Additionally, Palomba et al. combined the natural fatty acid methyl palmitate with albumin to constitute a stable spherical NP capable of inducing macrophages into a dormant state and inhibiting their phagocytosis (91). The albumin acts as a structural support and methyl palmitate regulates the internalization ability of macrophages (91).

NPs inhibit inflammatory pathways. As an antioxidant, CeO2 NPs is biosafe and can effectively intervene in disease processes (155). CeO2NPs synthesized by biological and materials engineering effectively reduce the superoxide flux of the mitochondrial electron transport chain (METC) and plasma membrane nicotinamide adenine dinucleotide phosphate (NADPH) oxidase (NOX), which regulate the antioxidant activity of macrophages (69). Moreover, CeO2NPs reduce MAP kinase/NF-kB-mediated signaling pathway activation by attenuating LPS induced IKB- α dilapidation and the nuclear translocation of NF KB/p65 (25, 26, 108). Macrophages exposed to CeO2NPs show downregulation of LPS-induced cytokine release (IL-1β, IL-6, TNF-α, HMGB1) (26, 108). Cargo-free loaded immunomodulatory NPs (iNPs) can interact with macrophages to regulate inflammatory processes (110, 111, 156). Furthermore, it was shown that cargo-free loaded iNPs reduced LPS-induced NF-κB p65 and MAPK p38 activation (156). This immunomodulatory property of cargo-free loaded iNPs is converted to a survival advantage in a lethal dose of LPS-induced sepsis mouse model (111). Therefore, nanomaterials inhibit their phagocytosis and inflammatory pathway activation, which are used to inhibit the overactivation of macrophages in sepsis.

NPs promote macrophage phagocytosis. A study reported that multiwalled carbon nanotubes (MWCNTs) mediate the activation of alveolar and parenchymal macrophages by CD40 and CD80 upregulation (82). Additionally, MWCNTs were recognized by the T-cell immunoglobulin mucin 4 (Tim4) receptor of macrophages, induced activation of the macrophage NLRP3 inflammasome, and enhanced phagocytosis of macrophages (83).

NPs promote inflammatory pathway. Silica NPs, iron oxide NPs (IONPs), and PLGA NPs can mediate the secretion of TNF-α by macrophages, which are involved in proinflammatory processes (86, 157). Moreover, an emerging nanomaterial called carbon dots (CDs) can target macrophages in lung tissue and induce macrophage endoplasmic reticulum stress (87). After coculture, macrophages phagocytosed CDs to induce NLRP3 inflammasome activation and proinflammatory cytokine secretion, which was proven by increased IL-1β and IL-8 (87). Additionally, polystyrene spheres denatured by amine treatment with a size of 60 nm can induce ROS production in macrophages by 20 μg/ml (88). Moreover, a nano copolymer can be endocytosed by macrophages to induce ROS production (89).Together, NPs could play a critical immunomodulatory role in the immunosuppressed state of sepsis.

NPs promote macrophage reprogramming. Peled et al. designed drug-free amphiphilic polymeric NPs generated by the self-assembly of hydrolyzed galactomannan (hGM)-linked copolymers (95). The drug-free amphiphilic polymeric NPs can be recognized by macrophage surface receptors (e.g. lectin-like receptors) polarize M1 macrophages to the M2 macrophage, as confirmed by the downregulation of the M1 marker (CD80) and the upregulation of M2 markers (CD163 and CD206) (95). Zhao et al. constructed Fe3O4@C/MnO2 NPs, which show promising photothermal functions and magnetic and catalytic activities, and can be implemented to induce M2-type macrophages to polarize to M1-type macrophages (158).Therefore, active intervention of engineered NPs in the M1-M2 macrophage polarization process could be applied to sepsis therapies.

## NPs monitoring macrophage function

The high-throughput platform is a monitoring platform that can use the biosensor developed by engineers to achieve continuous monitoring of cell behavior (159, 160). Currently, developed sensors are used to monitor macrophage function (74, 75). Monitor macrophage immune status. NPs are recognized and bound to receptors on the cell surface, initiating phagocytosis by macrophages and finally forming phagosomes (161). Recently, Law et al. designed an optical nanosensor that feeds back information about the environmental pH by monitoring changes in the Raman spectrum of p-mercaptobenzoic acid (p-MBA) to probe macrophage phagosome function (74). The optical nanosensor (p-MBA-NP) uses p-MBA-functionalized AuNPs as material and measures pH in macrophage phagosomes, which can be measured by changes in Raman spectra caused by the response of carboxyl groups to hydrogen ion concentrations in the environment, representing a new and precise means to evaluate macrophage function (74).

Monitor macrophage immunotherapy effect. Nanotechnology enables real-time monitoring of the physicochemical properties of macrophages and is used to observe the response to immunotherapy. For instance, a noninvasive imaging nitric oxide (NO) nanodetector allows real-time monitoring for macrophage immunotherapy (75). The detector promotes the assembly of NO imaging probes with colipids to construct a NO nanoreporter (NO-NR) liposome NP system, which monitors NO production during M1-M2 polarization in real time, reflecting the macrophage immunotherapeutic response (75).

Monitor macrophage temporal and spatial location. Apart from observation for therapeutic effect, marking macrophage locations is of great importance (162). As early as 2010, Wong et al. implemented nanomaterials for sepsis monitoring based on the phagocytosis of macrophages through quantitative susceptibility mapping (QSM) magnetic resonance (MRI) imaging to quantify iron (106). Once “Feridex”, a class of superparamagnetic iron oxide NP contrast agents, enters the body, they will be rapidly swallowed by Kupffer cells. Quantification of “Feridex” taken up by Kupffer cells by QSM MRI and linking the result to the immune response in sepsis progression may enable monitoring sepsis status (106). In addition, spherical dextran NP 64Cu-Macrin assembled from nontoxic polydextrose is used as a nanotracer for positron emission tomography (PET) for quantitative noninvasive assessment of cardiocpulmonary macrophages. This nanotracer can be used to investigate the spatiotemporal dynamics of macrophages in sepsis and act as an imaging biomarker for macrophages (**Figure 5**) (105). In 2021, Raja et al. developed chemically modified cellulose nanocrystal (CNC) derivatives by covalently linking PEGylated biotin and a perylene diimide (PDI)-based near-infrared organic dye to label and image J774A.1 macrophages in a dose-dependent manner, which realize the monitoring macrophage localization (76).

Therefore, nanomaterials could not only detect the localization of macrophages for the determination of tissue and organ damage severity but also assess the functional status of macrophages, which shows broad potential in the study and real-time monitoring of sepsis.

## Challenges and prospects of nanomedicine in sepsis

As a life-threatening pathophysiological syndrome, sepsis has a complex pathogenesis, in which the involvement of macrophages is particularly critical. The complicated pathophysiology of sepsis changes the phenotype and function of macrophages and induces macrophage exhaustion. At present, the drugs targeting macrophage function and the detection of macrophage function are still insufficient (4).Nanomaterials are promising candidates for targeting macrophages in sepsis. As drug carriers, NPs encapsulate and sequester active ingredients, enhancing macrophage-targeted time and specific delivery and/or improving drug biodistribution and/or bioavailability. In addition, nanomaterials modified by supramolecular structures can modulate macrophage function (17). For example, NPs can inhibit/enhance macrophage phagocytosis (90, 91) and suppress/promote its inflammatory pathways and cytokines secretion (82–84, 87, 89, 92, 93, 109, 163). NPs mediate M1-2 macrophage reprogramming as well (164, 165). Therefore, nanomaterials have the potential to treat macrophage-associated diseases, especially sepsis. Except for treatment, numerous clinical monitoring technologies of NPs are emerging, such as electrochemical and immunosensors for identifying infections, organ dysfunction, and immune dysregulation state (51, 166, 167). Detecting the localization of macrophages *via* nanomaterials can determine the severity of organ and tissue damage, thereby monitoring the progression of various macrophage-related diseases in real time (76). Although indirectly recognizing the pro-/anti-inflammatory cytokines’ lack of specificity, it provides directable roles in observing the inflammatory state of macrophage-associated diseases (51, 168–171). Furthermore, finding sepsis-specific biomarkers remains a legacy challenge. There is no doubt that the introduction of nanotechnology into preclinical studies in sepsis-associated macrophage therapeutics has made remarkable progress and has become a prospect for clinical applications.

Many challenges remain in this field. First, there is a lack of studies that have reported NPs targeting the epigenetic alterations of sepsis-associated macrophages. In LPS-stimulated macrophages, chromatin reorganization of enhancer regions was enhanced compared with that in resting macrophages (172, 173). The molecular mechanisms underlying the epigenetic regulatory effects of LPS include upregulation of the histone demethylase KDM6B *via* NF-κB initiation (8) and accumulation of histone deacetylase at the promoters of IL-1β and TNF, which lead to altered gene transcription (174). Thus, storing damaged macrophage function by regulating epigenetic alterations may be a hotpot. Second, a related study showed that intestinal microflora disruption may be harmful to macrophage phagocytosis promoting sepsis (175). Macrophages in lung tissues from gut microbiota-deficient mice show altered cellular responses and metabolic pathways (175), which also provides prospective for sepsis-managing gut microbiology. Third, macrophages produce extracellular traps (ETs) in response to various microorganisms and have similar characteristics to neutrophil ETs, which could be further explored in relation to nanomedicine (176). Fourth, metabolic changes in macrophages are also integral to the progression of sepsis. Moreover, stress erythrophagocytosis by the monocyte/macrophage system in the spleen could induce immunosuppression in sepsis *via* the STAT1 pathway (177). Consequently, there are many difficulties that can be further explored in the future. Furthermore, nanomaterials are widely applied for therapeutic interventions, but relatively few are designed to monitor macrophage function. Achieving effective monitoring of immune function in sepsis greatly guides subsequent treatment. Thus, the detection of the macrophage state needs to be achieved at a deeper level.

## Conclusion

To conclude, sepsis is a highly heterogeneous and clinically refractory syndrome. Based on the functional diversity and plasticity of macrophages, nanomedicine has achieved excellent breaks in the management of sepsis. However, the design of sepsis-state responsive nanotherapies interacting with the diversity and plasticity of macrophages is a clinical component that needs to be further explored. There is no question that the exploration process requires multidisciplinary collaboration among critical care medicine, immunology, molecular biology, biochemistry, pharmacology, and materials science.

## Author contributions

All authors listed have made a substantial, direct and intellectual contribution to the work, and approved it for publication. CS, JX, CG, WZ and XF wrote the review and designed the figures. YS revised the manuscript. All authors contributed to the article and approved the submitted version.

## Glossary

**Table:**

PAMPs	pathogen-associated molecular pattern molecules
LPS	lipopolysaccharides
PRRs	pattern recognition receptors
NPs	nanoparticles
PLGA	poly(lactide-co-glycolide)
DAMPs	damage-associated molecular pattern molecules
NF-**κ**B	nuclear factor-**κ**B
M**Φ**-NPs	macrophage-mimetic NPs
SPIO	superparamagnetic iron oxide
CK	compound K
ROS	reactive oxygen species
MAPK	mitogen-activated protein kinase
TLR4	toll-like receptor 4
CeO2NPs	cerium oxide NPs
METC	mitochondrial electron transport chain
NADPH	nicotinamide adenine dinucleotide phosphate
HMGB1	high mobility group box protein 1
iNOS	inducible nitric oxide synthase
eIF2α	eukaryotic initiation factor 2
COX-2	cyclooxygenase 2
MnO2	manganese oxide
MWCNTs	multiwalled carbon nanotubes
MPLA	monphosphatidyl lipid A
MDP	muramyl dipeptide
ALG	alginate
PHBV	poly(3-hydroxybutyrate-3-hydroxyvaleric acid)
SIRPα	signal-regulated protein-&alpha;
SHP-1	phosphatase-1
GSDMD	gasdermin D
LF	lactoferrin
DSF	disulfiram
hGM	hydrolyzed galactomannan
MMP-2	matrix metalloproteinase-2
COL	colchicine
CEP	cefadroxil
IL-1	interleukin-1
IL-6	interleukin-6
IL-18	interleukin-18
TNF-α	tumor necrosis factor α
TGF-β	transforming growth factor-β
p-MBA	p-mercaptobenzoic acid
CNC	cellulose nanocrystal
QSM	quantitative susceptibility mapping
MRI	magnetic resonance
PET	positron emission tomography
STAT	signal transducer and activator of transcription
ICAM-1	intercellular adhesion molecule 1
VCAM-1	vascular cell adhesion molecule 1
LFA-1	lymphocyte function-associated antigen 1
VLA-4	very late antigen 4
